# Comparative study on the clinical characteristics of local cases of COVID-19 and imported cases from abroad: A retrospective cohort study

**DOI:** 10.1097/MD.0000000000026933

**Published:** 2021-08-27

**Authors:** Jing-He Liu, Yu-Fei Chang, Shan-Fang Ma, Ling-Hang Wang

**Affiliations:** a Department of Emergency; b Department Of Infectious Disease Emergency, Beijing Ditan Hospital Capital Medical University, Beijing, China.

**Keywords:** clinical characteristics, COVID-19, disease presentation, imported cases

## Abstract

It is presently unknown whether imported cases of the 2019 coronavirus disease (COVID-19) have different characteristics when compared with local cases. To compare the clinical characteristics of local cases of COVID-19 in China compared with those imported from abroad.

This was a retrospective study of confirmed cases of COVID-19 admitted at the Beijing Ditan Fever Emergency Department between February 29^th^, 2020, and March 27^th^, 2020. The clinical characteristics of the patients were compared between local and imported cases.

Compared with local cases, the imported cases were younger (27.3 ± 11.7 vs. 43.6 ± 22.2 years, *P* < .001), had a shorter interval from disease onset to admission (1.0 (0.0–2.0) vs 4.0 (2.0–7.0) days, *P* < .001), lower frequencies of case contact (17.4% vs 94.1%, *P* < .001), fever (39.1% vs 82.4%, *P* < .001), cough (33.3% vs 51.0%, *P* = .03), dyspnea (1.9% vs 11.8%, *P* = .01), fatigue (7.5% vs. 27.5%, P = 0.001), muscle ache (4.7% vs. 25.5%, P < 0.001), and comorbidities (*P* < .05). The imported cases were less severe than the local cases, with 40.4% versus 5.9% mild cases, 2.8% versus 15.7% severe cases, and no critical cases (*P* < .001). The length of hospital stay was longer in imported cases than in local cases (32.3 ± 14.5 vs 21.7 ± 11.2 days, *P* < .001). The imported cases showed smaller biochemical perturbations than the local cases. More imported cases had no sign of pneumonia at computed tomography (45.0% vs 14.9%, *P* = .001), and none had pleural effusion (0% vs 14.9%, *P* < .001).

Compared with local cases, the imported cases of COVID-19 presented with milder disease and less extensive symptoms and signs.

## 1 Introduction

The 2019 coronavirus disease (COVID-19) is a global pandemic of acute respiratory distress syndrome (ARDS) caused by a novel coronavirus (SARS-CoV-2).^[1,2]^ SARS-CoV-2 is a member of beta genus coronaviruses closely related to SARS-CoV.^[3]^ The outbreak was first identified in December 2019 in Wuhan, China.^[4,5]^ On August 3^rd^, 2020, nearly all countries were affected, with a total of 17,918,582 individuals who contracted the disease and 686,703 who died from it.^[6]^ The majority of cases are in adults,^[7]^ and the majority of deaths are in elderly patients or in patients with cardiovascular, respiratory, or coagulation comorbidities.^[2]^ The mean incubation time is 5.2 days in Wuhan, China.^[8]^ The common signs of COVID-19 include fever, cough, and shortness of breath.^[2]^ acute respiratory distress syndrome and sepsis were reported in 100% of the patients with confirmed COVID-19 who died.^[9–12]^ There is no specific antiviral treatment for COVID-19 yet,^[2]^ but vaccines are available and show a promising degree of protection.^[13,14]^ Supportive care may help relieve symptoms and should include support of vital organ functions in severe cases.

Individuals may contract the disease locally in their home area or contract it while traveling and then importing it to their home area. Most of the cases at the start of the outbreak in a specific area are from international importation.^[15]^ A study suggested that countries with highly efficient screening capacities, such as Singapore, would detect as much as 2.8 times more imported cases than local ones.^[16]^ Nevertheless, most imported cases in Singapore were the early phase of the pandemic and contributed little to the later phases.^[17]^ The only effective way to prevent importation is to close the borders, but it does not prevent the patients who already imported the disease, nor the essential travelers who might be affected.^[15]^ Quarantine remains the best option for travelers.^[18]^

Imported cases refer to patients who caught COVID-19 in other countries than their country of residence, that is, including visitors from other countries and local resident returning home after being infected abroad. This is opposite to local cases, which refer to individuals who caught COVID-19 in their country of residency. It is presently unknown whether imported cases have different characteristics when compared with local cases. One study from Singapore reported that imported cases were identified more quickly.^[17]^ This might be an important issue for the management of the disease, especially in the context of a progressive end to confinement in many geographical areas. In addition, the imported, unlinked, and asymptomatic cases could be an important source of secondary contagion and amplification of transmission during confinement.^[17]^

Therefore, the aim of the present study was to compare the clinical characteristics of local cases of COVID-19 in China compared with those imported from abroad.

## 2 Methods

### 2.1 Study design and patients

In this retrospective study, we investigated the confirmed cases of COVID-19 admitted at the Beijing Ditan Fever Emergency Department between February 29^th^, 2020, and March 27^th^, 2020. All patients were confirmed of COVID-19 according to the WHO interim guidance.^[19]^ According to the source of the cases, all patients were divided into 2 groups: local cases and imported cases from abroad.

The study was approved by the Ethics Committee of Beijing Ditan Hospital affiliated with Capital Medical University. This article was a retrospective analysis, and the Committee waived the need for individual consent.

### 2.2 Laboratory tests

All suspected patients were admitted to quarantined observation rooms in the emergency department. A nucleic acid amplification test was performed on swab specimens from all patients with suspected disease at admission.^[19]^ The patients with a positive diagnosis were admitted to the hospital, whereas patients with an initial negative result were kept in quarantine and underwent a second nucleic acid test after 24 hours; of these, patients with a second negative result on the nucleic acid test were considered to not have an infection and were discharged from the hospital.

All confirmed cases underwent a blood gas analysis from arterial blood sampled from the radial artery by a physician. The electrode method was used for blood gas analysis on a Cobas B221 blood gas analyzer (Roche Diagnostics, Basel, Switzerland). Peripheral venous blood was also sampled routinely for the analysis of CD4^+^ and CD8^+^ peripheral blood mononuclear cells on a FACSCanto II flow cytometry system (BD Diagnostics, Sparks, MD). Pulmonary lesions were examined using a 16-row emotion computed tomography scanner (Siemens, Erlangen, Germany).

### 2.3 Data collection

Age, sex, complications (diabetes, liver diseases, high blood pressure, heart diseases, tumors, renal insufficiency, cerebrovascular diseases, rheumatic autoimmune diseases, lung diseases, and blood system diseases), the time from disease onset to admission, the time from visit to the first positive nucleic acid test, body temperature at admission (the body temperature measured by the physician in our hospital for the first time), close contact history (whether there was any history of contact with confirmed or suspected patients), clinical symptoms (fever (body temperature ≥37.3°C), cough, sore throat, headache, dyspnea, fatigue, muscle aches, and diarrhea), and clinical typing (based on the “Diagnosis and Treatment Program for Novel Coronavirus Pneumonia (Seventh Edition)” issued by the National Health Commission.^[20]^

### 2.4 Statistical analysis

The Kolmogorov-Smirnov test was used to confirm whether the continuous variable conformed to the normal distribution. The continuous variables conforming to the normal distribution were described as means ± standard deviations and were analyzed using the independent sample *t* test. The continuous variables that did not conform to the normal distribution were described as medians (interquartile ranges) and analyzed using the Mann-Whitney *U* test. The categorical data were described as numbers (percentages) and analyzed using the *χ*^2^ test or Fisher exact probability test, as appropriate. SPSS 22.0 (IBM, Armonk, NY) was used for all analyses. Two-sided (except for the *χ*^2^ test) *P* values <.05 were considered statistically significant.

## 3 Results

### 3.1 Characteristics of the patients

A total of 160 patients were confirmed with COVID-19 during the study period, including 109 imported cases and 51 local cases. Table 1 presents the characteristics of the patients. Compared with local cases, the imported cases were younger (27.3 ± 11.7 vs 43.6 ± 22.2 years, *P* < .001), had a shorter interval from disease onset to admission (1.0 [0.0–2.0] vs 4.0 [2.0–7.0] days, *P* < .001), showed a shorter time from admission to a first positive DNA test (1.0 [1.0–1.0] vs 1.0 [1.0–2.0], *P* < .001), a lower first measurement of body temperature (37.0 ± 0.7 vs 37.7 ± 0.7, *P* < .001), and a lower frequency of a history of contact with a confirmed/suspected case (17.4% vs. 94.1%, P < 0.001). Regarding the symptoms, compared with local cases, the imported cases had lower frequencies of fever (39.1% vs 82.4%, *P* < .001), cough (33.3% vs 51.0%, *P* = .03), dyspnea (1.9% vs 11.8%, *P* = .01), fatigue 7.5% vs 27.5%, *P* = .001), and muscle ache (4.7% vs 25.5%, *P* < .001). The imported cases also had lower frequencies of comorbidities (diabetes, heart diseases, hypertension, and lung diseases; all *P* < .05) compared with local cases. Of note, the imported cases were less severe than the local cases, with 40.4% versus 5.9% mild cases, 2.8% versus 15.7% severe cases, and no critical cases (*P* < .001). The length of hospital stay was longer in imported cases than in local cases (32.3 ± 14.5 vs 21.7 ± 11.2 days, *P* < .001).

**Table 1:** Characteristics of the patients.

Variables	Total (n = 160)	Imported cases from abroad (n = 109)	Local cases (n = 51)	*P*
Sex				
Female, n (%)	91 (56.9)	64 (58.7)	27 (52.9)	.492
Male, n (%)	69 (43.1)	45 (41.3)	24 (47.1)	
Age, y	29 (22–41.5)	24 (20–37)	42 (33–58)	<.001
Time from disease onset to visit, days	1.0 (1.0–3.0)	1.0 (0.0–2.0)	4.0 (2.0–7.0)	<.001
Time from visit to first positive nucleic acid test, days	1.0 (1.0–1.0)	1.0 (1.0–1.0)	1.0 (1.0–2.0)	<.001
Body temperature (°C)	37.05 (36.6–37.8)	36.8 (36.5–37.4)	37.8 (37.3–38.2)	<.001
Close contact history, n (%)	67 (41.9)	19 (17.4)	48 (94.1)	<.001
Clinical symptoms, n (%)				
Fever	83 (53.2)	41 (39.1)	42 (82.4)	<.001
Cough	62 (39.0)	36 (33.3)	26 (51.0)	.033
Sore throat	26 (16.7)	20 (19.1)	6 (11.8)	.252
Headache	15 (9.6)	8 (7.6)	7 (13.7)	.251
Dyspnea	8 (5.1)	2 (1.9)	6 (11.8)	.014
Fatigue	22 (13.9)	8 (7.5)	14 (27.5)	.001
Muscle aches	18 (11.4)	5 (4.7)	13 (25.5)	<.001
Diarrhea	2 (1.3)	1 (0.9)	1 (2.0)	.543
Complications, n (%)				
Diabetes	7 (4.4)	2 (1.8)	5 (9.8)	.034
Liver disease	1 (0.6)	0	1 (2.0)	.319
Heart diseases	3 (1.9)	0	3 (5.9)	.031
Hypertension	12 (7.5)	3 (2.8)	9 (17.65)	.002
Tumors	1 (0.6)	0	1 (2.0)	.319
Renal insufficiency	1 (0.6)	0	1 (2.0)	.319
Cerebrovascular diseases	3 (1.9)	2 (1.8)	1 (2.0)	1.000
Rheumatic autoimmune diseases	1 (0.6)	0	1 (2.0)	.319
Lung diseases	7 (4.4)	2 (1.8)	5 (9.8)	.034
Blood system diseases	1 (0.6)	0	1 (2.0)	.319
Clinical typing				<.001
Mild	47 (29.4)	44 (40.4)	3 (5.9)	
Ordinary	98 (61.3)	62 (56.9)	36 (70.6)	
Severe	11 (6.9)	3 (2.8)	8 (15.7)	
Critical	4 (2.5)	0	4 (7.8)	
Outcomes				
Length of hospital stay (days)	29 (19–42)	30 (21–44)	26 (15–38)	<.001
Death, n (%)	0	0	0	—
Mechanical ventilation, n (%)	0	0	0	—
ICU admission, n (%)	2 (1.3)	2 (1.8)	0	—

ICU = intensive care unit.

### 3.2 Laboratory tests

Table 2 presents the biochemical parameters of the patients. Compared with the local cases, the imported cases had lower Sjogren Syndrome A (Ro) (10.1 [3.1–27.8] vs 35.4 [5.0–200.9] U, *P* = .006), erythrocyte sedimentation rate (11.0 [6.0–16.0] vs 21.0 [10.0–39.0], mm/h, *P* < .001), C-reactive protein (1.4 [0.5–3.8] vs 12.9 [1.4–33.9] mg/dL, *P* < .001), higher platelets (240.61 ± 74.50 vs 214.19 ± 74.04  × 10^12^/L, *P* = .04), lower d-dimer (0.2 [0.1–0.3] vs 0.4 [0.3–0.7] mg/L, *P* < .001), lower alanine transaminase (19.7 [13.2–29.3] vs 25.5 [16.1–33.1] U/L, *P* = .04), higher albumin (45.50 ± 5.31 vs 41.49 ± 6.10 g/L, *P* < .001), lower lactate dehydrogenase (210.63 ± 72.39 vs 250.42 ± 95.63 U/L, *P* = .01), lower creatinine kinase-MB (15.92 ± 4.48 vs 19.54 ± 12.59 U/L, *P* = .050), higher CD4+ cells (736.56 ± 286.37 vs 566.68 ± 343.06 cells/mm^3^, *P* = .003), higher CD8+ cells (537.94 ± 258.58 vs 403.68 ± 306.47 cells/mm^3^, *P* = .01), lower blood pH (7.38 ± 0.04 vs 7.41 ± 0.05, *P* < .001), and higher partial pressure of carbon dioxide (PCO_2_) (5.67 ± 0.67 vs 4.90 ± 0.99, *P* < .001).

**Table 2:** Laboratory tests.

Variables	Total (n = 160)	Imported cases from abroad (n = 109)	Local cases (n = 51)	*P*
SSA, U	13.4 (3.4–56.4)	10.1 (3.1–27.8)	35.4 (5.0–200.9)	.006
ESR, mm	13.0 (7.0–21.0)	11.0 (6.0–16.0)	21.0 (10.0–39.0)	<.001
CRP, mg/dL	2.2 (0.7–13.0)	1.4 (0.5–3.8)	12.9 (1.4–33.9)	<.001
White blood cell count (×10^9^/L)	5.33 (4.41–6.905)	5.34 (4.51–6.69)	5.06 (4.345–7.005)	.641
Neutrophil count (×10^9^/L)	3.155 (2.12–4.19)	3.17 (2.14–4.13)	3 (2.1–4.225)	.629
Lymphocyte count (×10^9^/L)	1.635 (1.13–2.05)	1.73 (1.33–2.14)	1.29 (0.995–1.77)	.650
Monocyte count (×10^9^/L)	0.36 (0.255–0.47)	0.39 (0.27–0.5)	0.31 (0.21–0.4)	.564
Eosinophil count (×10^9^/L)	0.02 (0.01–0.08)	0.03 (0.01–0.09)	0.01 (0–0.04)	.717
Hemoglobin, g/L	142 (130–151.5)	143 (131–152)	140 (125.5–150)	.151
Platelets, (×10^12^/L)	224 (181.5–263.7)	232 (194–268)	208 (152–256)	.038
PT, s	12.1 (11.6–12.6)	12 (11.5–12.6)	12.1 (11.9–12.9)	.587
APTT, s	32.25 (30.3–33.95)	32.3 (30.4–33.9)	31.5 (27.5–33.85)	.196
d-dimer, mg/L	0.3 (0.2–0.4)	0.2 (0.1–0.3)	0.4 (0.3–0.7)	<.001
ALT, U/L	20.8 (14.1–31.2)	19.7 (13.2–29.3)	25.5 (16.1–33.1)	.043
AST, U/L	21.6 (17.3–30.5)	20.8 (17.3–27.8)	22.9 (17.3–42.9)	.302
Albumin, g/L	45 (42.35–47.4)	45.6 (43.6–47.7)	42 (36.2–45.9)	<.001
Creatinine, μmol/L	66.6 (52.85–79.7)	65.8 (53–79.9)	67.4 (51.35–78.35)	.465
Urea nitrogen, mmol/L	4.225 (3.61–5.045)	4.27 (3.71–5.06)	4.15 (3.46–4.955)	.280
Lactate mmol/L	2.02 ± 0.53	2.05 ± 0.50	1.94 ± 0.61	.270
LDH, U/L	195 (167.4–249.55)	186 (165.4–231.9)	232.3 (184.9–315.9)	.010
CK, U/L	81.3 (60.9–125.4)	81.5 (61.8–122.1)	79.8 (50.1–130.0)	.662
CKMB, U/L	15.4 (12.9–19.35)	15 (13–18.1)	15.8 (12.65–21.55)	.050
CD4, cells/mm^3^	640 (462–858.5)	706 (520–919)	479 (343.5–690.5)	.003
CD8, cells/mm^3^	446 (307.5–655.5)	498 (349–659.5)	328.5 (175–491)	.010
CD4/CD8	1.46 (1.12–1.815)	1.44 (1.1–1.76)	1.545 (1.16–2.16)	.119
pH	7.39 ± 0.05	7.38 ± 0.04	7.41 ± 0.05	<.001
PCO_2_, mmHg	5.39 ± 0.88	5.67 ± 0.67	4.90 ± 0.99	<.001
PO_2_, mmHg	13.19 (11.29–14.695)	13.6 (12.63–14.73)	11.545 (8.91–14.56)	.145
HCO_3_, mEq/L	24.2 (22.65–25.5)	24.1 (23.1–25.5)	24.25 (21.7–25)	.123

ALT = alanine transaminase, APTT = activated partial thromboplastin time, AST = aspartate transaminase, CK = creatinine kinase, CRP = C-reactive protein, ESR = erythrocyte sedimentation rate, LDH = lactate dehydrogenase, PCO_2_ = partial carbon dioxide pressure, PO_2_ = partial oxygen pressure, PT = prothrombin time, SSA = Sjogren's syndrome A (Ro).

### 3.3 Imaging examination

Table 3 presents the results of the imaging evaluations. Compared with the local cases, more imported cases had no sign of pneumonia at computed tomography (45.0% vs 14.9%, *P* = .001), none had pleural effusion (0% vs 14.9%, *P* < .001), and fewer had ground-glass shadow (44.0% vs 76.6%, *P* < .001).

**Table 3:** Imaging examinations.

Variables	All (n = 160)	Imported cases from abroad (n = 109)	Local cases (n = 51)	*P*
Pneumonia, n (%)				
None	56 (35.9)	49 (45.0)	7 (14.9)	.001
Unilateral	35 (22.4)	24 (22.0)	11 (23.4)	
Bilateral	65 (41.7)	36 (33.0)	29 (61.7)	
Pleural effusion, n (%)	7 (4.5)	0	7 (14.9)	<.001
Nodule, n (%)	21 (13.5)	17 (15.6)	4 (8.5)	.234
Ground-glass shadow, n (%)	84 (53.9)	48 (44.0)	36 (76.6)	<.001

## 4 Discussion

It is presently unknown whether imported cases of COVID-19 have different characteristics when compared with local cases. Therefore, this study aimed to compare the clinical characteristics of local cases of COVID-19 in China compared with those imported from abroad. The results suggest that compared with local cases, the imported cases of COVID-19 presented with milder disease and less extensive symptoms and signs.

In the present study, the imported cases were admitted to the hospital earlier after symptom onset than the local cases. This phenomenon was also observed in Singapore, where the screening protocols are highly efficient.^[16,17]^ Since the present study examined patients mainly admitted in March, that is, well after the start of the pandemic and the implementation of sanitary measures, this could denote that patients coming back from travel might be more aware than locals of the possibilities of COVID-19 transmission and could consult earlier after symptom onset, as instructed by public health authorities. In addition, most of them would be in quarantine and aware of the necessity of reporting symptoms. A meta-analysis observed that higher disease awareness could lead to a milder presentation.^[21]^ The knowledge about red flag symptoms, which is provided to anyone entering quarantine for COVID-19 prevention, results in better management.^[22]^

Many patients were asymptomatic or presymptomatic at admission, as supported by a study reporting 31% to 56% of such patients.^[23–26]^ This resulted, at presentation, in milder disease, fewer biochemical abnormalities, and fewer imaging abnormalities compared with local cases. Because of border surveillance and mandatory testing, the patients can be identified many days before the onset of symptoms, whereas many local cases are tested when the symptoms appear. Therefore, many patients who would be asymptomatic or with non-specific mild symptoms were hospitalized. In addition, because all inbound individuals are told to check for specific symptoms, they might consult earlier than local patients. Although the imported cases are milder, an earlier discovery leads to longer hospitalization since they can be discharged after there is no risk of virus transmission, as for local cases. Supporting this, 2 imported cases had to be admitted to the ICU during hospitalization. Of note, none among the 160 patients required mechanical ventilation or died. This is a better prognosis than what is reported in the literature. Indeed, a study reported an overall in-hospital mortality of 15% to 20%, with up to 40% requiring admission to the ICU.^[2]^ On the other hand, a cohort study showed that the overall mortality to COVID-19 was 2.3% in China, but with higher mortality rates in the older age groups.^[7]^ The good prognosis observed in the present study could be because the patients were relatively young (32.5 ± 17.5 years overall). In addition, the imported cases were younger than the local cases. The imported cases were more likely to be workers or students, that is, individuals in the active population, whereas the local cases could cover the whole range of the local population. Still, it is only a hypothesis because the actual age of all people who crossed the border during the study period is unknown and cannot be compared to the age of the local population. Nevertheless, the local cases had higher frequencies of comorbidities (diabetes, heart diseases, hypertension, and lung diseases) known to negatively influence the disease outcomes,^[10–12,27]^ but they nevertheless did not fare worse.

This study has limitations. It was a retrospective study in a small sample of patients from a single hospital. Only the characteristics at admission were examined. In addition, because of the retrospective nature of the study, only the data that were routinely measured and recorded in the charts could be analyzed. Additional studies are still necessary to refine the results of the present study.

In conclusion, the results suggest that compared with local cases, the imported cases of COVID-19 presented with milder disease and less extensive symptoms and signs, possibly because of mandatory testing and higher awareness of symptoms. Those results suggest a higher surveillance and/or disease awareness in patients who might have been infected abroad.

## Acknowledgments

The authors thank all participants who contribute for this manuscript.

## Author contributions

**Data curation:** Jinghe Liu.

**Investigation:** Yufei Chang.

**Methodology:** Jinghe Liu.

**Software:** Yufei Chang.

**Writing – original draft:** Jinghe Liu.

**Writing – review & editing:** Shanfang Ma, Linghang Wang.
